# Collaborative innovation in sustainable fashion: A consumer-centric perspective on adoption and brand performance

**DOI:** 10.1371/journal.pone.0337902

**Published:** 2026-06-11

**Authors:** Sasichakorn Wongsaichia, Teerapong Pienwisetkaew, Chavis Ketkaew

**Affiliations:** 1 Center for Sustainable Innovation and Society, Khon Kaen University, Amphur Muang, Khon Kaen, Thailand; 2 International College, Khon Kaen University, Amphur Muang, Khon Kaen, Thailand; Thammasat University, THAILAND

## Abstract

Involving consumers in product ideation and development can generate innovations that better satisfy customer needs and, hence, are more likely to be adopted. Moreover, such customer-centric innovation processes reinforce the emotional as well as functional linkages between consumers and sustainable products, a dimension that is significantly more important than current collaboration at the firm level or in the supply chain for sustainability research. This study, therefore, explores how perceived collaborative innovation (PCI) affects perceived brand performance (PBP) in the sustainable fashion sector, particularly uncovering the key psychological mechanisms underlying how PCI affects consumer behavior. Specifically, this study reveals the mediating roles of the perceived benefits of sustainable fashion (PBSF) and consumer willingness to adopt sustainable fashion (CWASF) in the relationship between PCI and PBP. Using survey data of 602 Thai consumers (from an emerging economy), which can reflect the behavior of consumers in developing markets, and employing a Structural Equation Modeling (SEM) methodology, this study finds that PCI positively affects both PBSF and CWASF, leading to enhanced PBP. Moving beyond firm-level collaboration for sustainability by applying a consumer-centered perspective, the study introduces a framework that identifies the linkages between innovation perception, behavior, and brand performance. By demonstrating how collaborative innovation is a psychological driver of sustainable adoption and brand competitiveness, this study provides insights to help accelerate sustainability in the fashion industry, particularly in emerging markets.

## 1. Introduction

The fashion industry has a notorious impact on the environment [1]. It produces enough carbon emissions to destroy the planet. The production of fashion includes the use of toxic pesticides and the release of huge amounts of plastic waste into the world’s oceans, while also using excessive amounts of water to dye and wash garments, polluting rivers and threatening the health of local communities. According to the United Nations Environment Programme (UNEP), the fashion industry is accountable for 2% to 8% of global greenhouse gas emissions, in addition to producing 9% of annual ocean microplastic pollution [2]. Production patterns are often driven by the rapid turnover of trends, which fast fashion magnifies by providing low-cost, high-volume fashion goods that are disposed of after a brief period of time [3]. Growing concern about the environmental, social, and economic sustainability of the apparel industry is fuelling demands for sustainable fashion — apparel that is produced sustainably, is consumed responsibly, and addresses the global social and environmental concerns connected to the way garments are made, distributed, consumed, and discarded. Sustainable fashion goods are also described using other terms, including eco-fashion, green fashion, ethical fashion, organic clothing, circular fashion, and slow fashion [4,5].

Global concerns over environmental sustainability, coupled with the rapid growth of fashion consumption and Thailand’s manufacturing sector, present a timely opportunity to explore how sustainability is practiced by fashion consumers. Sustainable fashion not only tackles environmental concerns but also reflects evolving modernity and changing identities of today’s consumers [6]. Moreover, sustainable fashion has become a key issue within Southeast Asia’s burgeoning textile industry. While the Thai apparel market is anticipated to reach USD 1,064 million in 2024 and sustain a compound annual growth rate (CAGR) of 12.62% from 2024 to 2029, potentially reaching USD 1,928 million by 2029, Thailand has been one of the leading exporters of apparel in Asia and is gradually mainstreaming sustainable practices in its manufacturing sector. Further, it posits that Thailand’s cultural context and patterns of consumption are unique to the region, acknowledging appreciation for community-based production and support for handmade craftsmanship [7]. Cultural narratives around sustainable consumption are also portrayed as reflective of the country’s modernity and changing values, specifically how sustainable consumption is perceived by young consumers. Ultimately, the Thai fashion industry is characterized by collaborative fashion practices that reflect community-based, creative-based, and environmentally-based approaches to sustainability.

Chesbrough (2003) [8] first proposed the Open Innovation Theory, focusing on the directions and consequences of external and internal knowledge flows for improving innovation performance. This paper, however, focuses on collaborative innovation, which refers to active partnerships or collaborations involving and connecting different stakeholders. Collaborative innovation with supply chain partners and customers is critical for gaining competitive advantages [9]. In particular, customer co-creation is critical since it involves integration of supply chain and product innovation processes to deliver enhanced value propositions, using tools like product life-cycle management systems [10,11]. The definition of Wang and Hu (2020) [12] highlights the involvement of supply chain partners in the innovation process; however, their description can be broadened to include customers who are vital for any collaborative innovation. This perspective is relevant and valuable, particularly in a retail setting with consumer-focused brand performance. It is especially relevant in the sustainable fashion industry, where innovations have to be aligned with sustainability-focused consumer preferences. Collaborative innovation in sustainable fashion can be operationalized through digital transformation, including integration of different platforms, exploitation of different data resources, and the process of sharing knowledge within and across different boundaries through iterative collaboration [13]. A local fashion project involved a group of fashion students in collaboration with a group of skilled garment makers from diverse cultural backgrounds to design and produce garments that were sold to support local community development through applying sustainability. The project’s success demonstrated collaborative innovation in practice by applying empathy, inclusivity, and knowledge-sharing to achieve sustainability. Studies have found that businesses that practice collaborative innovation better achieve their intended outcomes [14]. External knowledge is essential for gaining competitiveness in turbulent markets [13]. This is particularly relevant in the context of sustainable fashion, where collaborations have the potential to create innovations that resonate with sustainability-focused consumer preferences [15]. An example of this is the development of the eco-friendly recycled cotton-polyester blend used in denim manufacturing by Pranta et al. (2024) [16]. In the context of sustainable organizational performance, collaborative innovation can result in economic, social and environmental advantages [9]. Customer and stakeholder involvement in the innovation processes has emerged as a new way to foster sustainable value co-creation and build innovation ecosystems [10,17]. This way of engaging employees, local communities, suppliers, and policymakers through involvement, dialogue, and collaboration—within and between organisations—is increasingly relevant in the fashion industry.

Prior research has consistently demonstrated that collaborative innovation enhances organizational and innovation performance, primarily from a firm-centric perspective focusing on operational efficiency and financial outcomes [18–20]. However, this perspective overlooks how collaborative innovation is interpreted and evaluated by consumers, particularly in sustainability-driven markets where consumer values play a central role. Despite the growing emphasis on sustainability, the relationships between perceived collaborative innovation (PCI) and perceived brand performance (PBP), particularly through the mediating roles of perceived benefits (PBSF) and consumer willingness to adopt (CWASF), remain largely unexplored in the sustainable fashion context [12,19,21]. This gap is particularly important in the sustainable fashion industry, where brand success increasingly depends on consumers’ perceptions of value, ethical alignment, and behavioral intentions rather than purely financial metrics. Without understanding these mechanisms, existing research provides an incomplete explanation of how collaborative innovation translates into market outcomes. To address this limitation, the present study develops a consumer-centric model that examines the direct and indirect effects of PCI on PBP, incorporating PBSF and CWASF as mediating mechanisms. By doing so, this research extends open innovation theory into the consumer domain and provides a more comprehensive understanding of how collaborative innovation drives sustainable brand performance. To investigate these relationships, this study aims to examine the direct and indirect effects of PCI on PBP in Thailand’s apparel industry. Specifically, the study has two primary objectives:

**Objective 1:** To assess the direct impact of PCI on PBSF, CWASF, and PBP, thereby determining the strength and significance of these relationships.

**Objective 2:** To examine how PBSF and CWASF mediate the relationship between PCI and PBP.

This analysis explores the relationship between PCI and consumer engagement, interpreted as the realization of PBSF and CWASF, and subsequently how this affects the PBP of sustainable fashion brands. The results of this study offer practical business insights for sustainable fashion enterprises seeking to connect with consumers and utilise innovation to optimise market performance.

This part of the log frames the upcoming sections of the paper. It describes the sections on literature review and hypothesis development and sets out the theoretical framework on the direct and indirect effects of collaborative innovation in sustainable fashion. It then outlines the methodology, followed by the results, and discusses these. The implications for theory and practice follow, leading to suggestions for future research. The conclusions summarise the main findings of the paper.

## 2. Literature review and hypothesis development

This section outlines the theoretical foundations and key concepts that guide the study. It begins by defining PBP and then examines how PCI shapes PBP within the sustainable fashion industry, highlighting the mediating role of consumer engagement. Each hypothesis is grounded in its relevant theoretical background, leading to a coherent research framework centered on consumer perspectives.

### 2.1. Perceived Brand Performance (PBP)

Perceived brand performance (PBP) is concerned with how consumers evaluate a brand’s overall performance in terms of providing value to them and others [22]. This concept is particularly relevant in sustainability-driven markets where non-financial aspects such as consumer loyalty, satisfaction, trust, and, indeed, the reputation of a brand and its social responsibility are becoming key performance indicators of brand success in addition to sales growth and profitability [23]. Consumer performance of a brand is also about how consumers perceive and interact with a brand, i.e., a brand’s market presence in terms of awareness, attitude, and knowledge amongst its target market. In relation to fast fashion, further brand-related factors that may contribute to a consumer’s PBP include their attitudes and awareness towards sustainable fashion. In the sustainable fashion market, PBP is a multi-dimensional construct that comprises: consumers’ perceptions of value (market presence and purchasing experience/satisfaction) and customer loyalty [23,24].

PBP could be one of the innovative lenses through which collaborative innovation is perceived by both customers and the market, as reflected in their perceptions of brand performance (PBP). While previous studies have succeeded in depicting how collaborative innovation could bring better organizational performance [25]. The majority of them are only focused on how collaboration could improve performance within organizations in terms of the company’s operational and financial performance. Indeed, there is also limited evidence showing the extent to which collaborative innovation strategies such as co-design, stakeholder engagement, and innovative sustainable materials could foster consumers’ PBP. However, recent studies found that in perceived buying plus performance (PBP), innovation containing ethics and sustainability values is more appreciated by consumers [26]. For instance, innovative next-generation textile-based products, blockchain technology for traceability, and trust [25], and others could enhance consumers’ perception towards positive change that could intensify brand performance.

Providing a deeper insight into PBP, this study examines how key consumer-centric measures of loyalty, satisfaction, and reputation are influenced by collaborative innovation in sustainable fashion. Consumers’ evaluations of sustainable brands extend beyond perceived value offered by the product or service to assessments of ethical sourcing, innovative identity, and marketing authenticity [27]. Long-term performance of sustainable brands is consequently enhanced through collective efforts. This study thus strengthens the theoretical contribution of the research by extending the applicability of innovation theory into the consumer domain and provides insights into how collaborative innovation can enhance the competitiveness of fashion firms and reinforce positive brand image in a sustainable market environment.

### 2.2. Perceived Collaborative Innovation (PCI)

PCI refers to the consumers’ perception that sustainable fashion brands actively engage in collaboration with other companies, adopt external ideas to improve products and services, and co-create with their customers as part of the innovation process [12,28]. On the other hand, it reflects consumers’ perception that a sustainable fashion brand incorporates external knowledge sources into the innovation process within the organization [9,12]. Open Innovation Theory posits that accessing external knowledge sources is critical to enhancing innovation performance [8]. Swink (2006) extended this conceptualization by highlighting the strategic importance of integrating supply chain innovation and product innovation to create value for customers [29]. It suggested that firms employ a portfolio of product life-cycle management systems to sense and seize and reconfigure internal and external resources and partner with supply chain partners to facilitate product and process innovation and create value for customers. Cheng et al. (2024) [18] built on this stream of literature and developed a dynamic capabilities framework to underscore the importance of sense, seize, and reconfigure opportunities by involving suppliers, customers, and other stakeholders in innovation processes. Naeem et al. (2026) [17] further supported this holistic approach to collaborative and open innovation by highlighting the impact of co-creation, co-design, and involving civil society and user communities in the innovation process in building wider innovation networks that generate ideas for sustainable innovation. Thus, PCI reflects consumers’ perception of a brand’s external collaboration and co-creation practices within its overall innovation efforts.

#### 2.2.1. PCI and Perceived Benefits of Sustainable Fashion (PBSF).

For sustainable fashion brands to achieve their goals, it is not enough to internalize knowledge; they must also externalize by involving partners such as suppliers, stakeholders, and customers, thereby co-creating value that is environmentally, socially, and economically significant to customers [30]. Collaboration with customers through cooperation and interaction also helps sustainable fashion brands to meet consumer expectations of sustainability by creating value in the form of relevant products and desirable experiences [31], thus enabling them to be more competitive and flexible to respond to rapid changes in the environment [32]. This study defines PBSF as consumers’ perception that sustainable fashion decreases environmental impact and supports socially responsible actions while saving long-term expenditure with economic benefits [33]. The concept is similar to consumer-perceived value, a psychological concept that refers to an individual’s perception of utility that he or she receives in exchange for the price paid through shopping, measuring overall value in terms of differences consumers believe exist between perceived benefits and perceived costs of retail products and services in evaluation [34,35]. By defining sustainable fashion from consumers’ perspectives and highlighting different aspects of their perception of sustainable fashion, the study provides a new construct and strengthens theory on consumer behavior by categorizing the environmental, social, and economic aspects of sustainable fashion from the consumers’ point of view as different types of consumer-perceived value [36].

H1: PCI positively influences PBSF.

#### 2.2.2. PCI and Consumer Willingness to Adopt Sustainable Fashion (CWSF).

In addition to promoting sustainable practices within the industry, PCI can also be applied to encourage CWASF. CWASF is demonstrated through brands that advocate for sustainability. The Theory of Planned Behavior (TPB) is one framework that explains the influence of Attitudes, Subjective Norms, and Perceived Behavioral Control on consumers’ intentions and behaviors toward sustainability [37]. Further, customer value co-creation in digital businesses influences a customer’s purchase decision [38]. PCI can be viewed through the lens of the Theory of Planned Behavior as well as the Diffusion of Innovations theory, with a focus on relative advantage, compatibility, complexity, and observability [39]. CWASF is typically measured in terms of consumers’ willingness to pay a higher price for sustainable fashion, preference for sustainable products over traditional products, and willingness to search for sustainable products [37,40,41]. A further predictor of innovation adoption is the perceived innovativeness of the product, which conveys the outstanding environmental attributes of the sustainable fashion product to potential customers [9]. PCI has a direct relationship with CWASF.

H2: PCI positively influences CWASF.

#### 2.2.3. PCI and PBP.

The integration of external knowledge and partnerships through PCI directly enhances PBP. Swink (2006) emphasizes how collaborative innovation integrates supply chain and product innovation efforts, enabling firms to remain competitive in dynamic markets. For sustainable fashion brands, PCI fosters innovations that resonate with consumer preferences for sustainability, leading to improved brand performance [12]. This is particularly the case because, in sustainability-driven markets, brand performance is increasingly assessed not just by sales or revenue, but also by non-financial metrics such as corporate social responsibility (CSR) credibility, which is directly influenced by a brand’s sustainability and ethical practices [42]. Research shows that PCI positively impacts non-financial aspects of PBP, including customer satisfaction, loyalty, and reputation [13]. Liu et al. (2020) [23] further emphasize that in sustainability-driven markets, such non-financial indicators—including trust, brand image, and corporate social responsibility credibility—are central to evaluating brand performance, as they capture consumer-based outcomes that extend beyond traditional financial measures. This emphasis on non-financial indicators reflects how values-driven consumers reward credible ethical alignment and co-creative practices (e.g., trust, legitimacy, identification), which typically precede and enable downstream financial returns [42,43]. Collaborative innovation allows brands to differentiate themselves in the market, thereby enhancing their PBP [44]. However, these benefits are not unconditional: excessive openness can generate diminishing returns and coordination costs, and risk knowledge leakage, thereby dampening brand performances [42]. Moreover, if collaborative claims outpace substantive practice, consumer skepticism can reduce CSR credibility and loyalty [13]. This is especially critical in sustainable fashion, where consumer loyalty hinges on meeting ethical and environmental standards.

H3: PCI positively influences PBP.

### 2.3. PBSF as perceived value

Perceived benefits of sustainable fashion (PBSF) refers to how consumers form psychological evaluations of sustainable fashion’s utility based on the balance of sustainable fashion’s benefits and costs. By paying particular attention to the environmental, social, and economic benefits of sustainable products (PBSF), such as sustainable materials and production practices, good working conditions for employees, fair payment for employees, and community contributions, sustainable fashion benefits play very important roles in consumers’ brand-value perceptions [40,45]. Communication of the PBSF (e.g., environmental and social benefits) through certifications, messages, labelling, and visual packaging (e.g., stamps, logos), as well as consumer experiences with a brand, can positively affect consumer engagement with the brand [33]. An application of perceived value theory, PBSF explicitly translates environmental and social utility into adoption intentions. For example, customers might perceive value if a brand states that its fashionable denim products are made of 100% organic cotton, manufactured under fair trade practices, and have long durability. Consumers are more likely to adopt sustainable fashion and exhibit brand loyalty when they become aware of the positive contribution that a brand makes through sustainable fashion [46]. Therefore, PBSF are key drivers of consumer behavior in sustainable fashion markets.

H4: PBSF positively influences CWASF.

### 2.4. CWASF as behavioral intention

Consumers’ behavior towards sustainable fashion influences a brand’s performance positively or negatively. The Theory of Planned Behavior explains that CWASF stems from the consumers’ attitudes and intentions towards sustainable fashion, affecting the performance of brands by generating customer value [37,47]. Furthermore, sustainability-oriented actions on the part of a brand are interpreted by customers as a sign of transparency and ultimately generate higher levels of customer satisfaction [48,49]. Innovative products, moreover, are developed for sustainable fashion that offer customers new options for individualization and thus strengthen loyalty [50]. This in turn leads to higher perceived benefits for both the brand and the retailer. From a marketer’s perspective, consumers’ willingness to pay a higher price for sustainable products (CWASF) can open up profitable market opportunities and gain legitimacy and visibility in the sustainable fashion market [51]. Thus, sustainable fashion products are seen by other customers as being suitable for adoption since they are compatible with consumers’ values. By communicating with their customers transparently about their actions to increase sustainability, fashion retailers generate trust [43,52,53]. Ultimately, customers regard sustainable-fashion-oriented brands as being more reputable than other retailers. Therefore, CWASF is a decisive predictor of PBP in the sustainable fashion sector.

H5: CWASF positively influences PBP.

### 2.5. Mediating effects of PBSF and CWASF

While perceived collaborative innovation (PCI) reflects firms’ ability to integrate external knowledge and co-create value in line with Open Innovation Theory [28,8]. Its impact on consumer outcomes does not occur automatically. Instead, the influence of PCI on consumer behavior and brand performance unfolds through internal cognitive and behavioral mechanisms, which are essential to explain how innovation is translated into market success. Importantly, without considering these mediating mechanisms, the relationship between PCI and perceived brand performance (PBP) would remain theoretically incomplete, as innovation alone cannot directly generate brand outcomes unless it is first interpreted and acted upon by consumers [9,18].

From a consumer behavior perspective, this mechanism works as a cognitive–behavioral process. Initially, PCI increases consumers’ perceptions of the environmental, social, and economic benefits of sustainable fashion. First, collaborative innovation signals transparency, credibility, and alignment with sustainability values. Therefore, it strengthens the perceived benefits of sustainable fashion (PBSF) [30,33]. These perceptions act as a cognitive evaluation mechanism that affects how consumers perceive the value of sustainable fashion. When consumers perceive higher benefits, they develop more favorable attitudes and intentions to adopt sustainable fashion [54].

Moreover, perceived benefits of PBSF translate into consumer willingness to adopt sustainable fashion (CWASF). Drawing on the lens of the Theory of Planned Behavior [37] and Diffusion of Innovations [51]. Behavioral intention is an important intermediate variable between perception and actual behavior. As long as consumers perceive clear advantages of sustainable fashion, feel that sustainable fashion is congruent with their values, and can experience sustainable fashion benefits [7,34,55]. They are more likely to adopt sustainable fashion. Consequently, PBSF is the critical mediator through which PCI influences CWASF.

Thirdly, to further reveal the mechanism through which PCI affects PBP, consumer willingness to adopt (CWASF) is identified as an important pathway. The stream of research focusing on consumer willingness to adopt sustainable fashion also supports this claim, as sustainability perception influences intentions and actions that, in turn, are manifested in consumer loyalty, repeat purchase, positive word of mouth, brand talk, and overall favorable brand evaluation [48,56]. Moreover, sustainable offerings enhance brand competition and performance as consumers are willing to pay a premium [34,55,57].

These findings reveal a cognitive–behavioral chain of effects that needs to be in place before perceived collaborative innovation (PCI) has an effect on perceived brand performance from a consumer perspective. The Stimulus–Organism–Response (S–O–R) framework suggests that the first step is cognitive internalisation of PCI (PCI has to be recognised by the consumer cognitively) followed by a behavioural dimension in which the cognitive internalisation is translated into consumer wrestling with artefacts and artefacts with self (PBSF) and ultimately how this cognitive and behavioural interpretation will translate into CWASF and consequently into PBPF [58–60].

In order to explain or moderate how and why perceived collaborative innovation (PCI) has effects on consumer-related outcomes and brand performance, mediation is conceptualized here. In this study, perceived benefits of sustainable fashion (PBSF) is treated as a cognitive mediator which explains why PCI has positive effects on consumer-related outcomes and brand performance. In contrast, consumer willingness to adopt sustainable fashion (CWASF) is treated as a behavioural mediator that explains why PCI has positive effects on consumer-related outcomes and brand performance. Importantly, PBSF and CWASF form a sequential mediation in which cognitive evaluation precedes behavioural intention, and ultimately affects perceived brand performance (PBP). Consequently, PCI does not have direct effects on brand performance, but effects are transmitted through internal consumer evaluations and consumer behaviour.

Therefore, PBSF and CWASF are always mediating variables in the relationship between PCI and PBP, either independently or sequentially [44,61].

H6: PBSF mediates the relationship between PCI and CWASF.

H7: CWASF mediates the relationship between PCI and PBP.

H8: PBSF and CWASF mediate the relationship between PCI and PBP.

### 2.6. Proposed research framework

**Fig 1:**
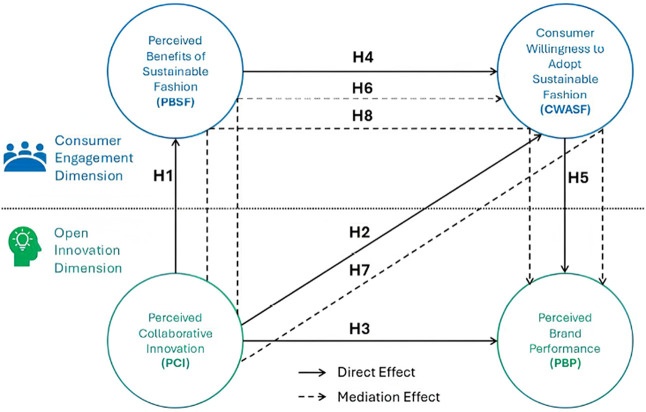
Proposed research framework and hypothesis.

Fig 1 illustrates the research framework. The independent variable, Consumer Initiated PCI, is posited to have a direct effect on the two sustainable fashion factors, PBSF and CWASF. Both the PBSF and CWASF are further posited to have a positive effect on PBP. Additionally, PCI is posited to have a direct positive effect on PBP through consumer engagement with the brand. The proposed framework captures the intricate relationships between the variables and highlights the pivotal role of collaborative innovation in driving consumer engagement and brand performance in the sustainable fashion industry [44,8,19]. The framework is further categorised into the Open Innovation Dimension, comprising PCI, PBP, and the Consumer Engagement Dimension, comprising PBSF and CWASF.

## 3. Methods

### 3.1. Variables and measures

The survey was a structured questionnaire with three sections: Screening Questions, Demographics, and Measurement Constructs (Appendix 1). The Screening Questions were used to screen the respondents to ensure that they were familiar with and had sufficient experience with sustainable fashion. Respondents were asked a series of questions, such as whether they are familiar with terms such as sustainable, green, organic, and eco-friendly, including the application in the context of fashion; familiar with practices that are deemed to be unethical and ethical practices relating to labor in the fashion apparel industry; have purchased products classified as sustainable fashion; or seriously considered purchasing such products. Respondents who answered “No” to the questions were instructed to complete the end of the survey.

The second section of the survey focused on gathering demographic information such as gender, age, level of education completed, annual income, and typical shopping habits. For the third section of the survey, the constructs of perceived collaborative innovation (PCI), perceived benefits of sustainable fashion (PBSF), consumer willingness to adopt sustainable fashion (CWASF), and perceived brand performance (PBP) were measured using a 10-point Likert-type scale (1 = Strongly Disagree, 10 = Strongly Agree).

The items for these four constructs were developed using a hybrid method of items from validated scales, reworded items, and additional items created based on the concepts and consumer-related insights from previous research studies.

For the PCI items, those relating to PCI were adapted from Wang and Hu (2020) [12], with appropriate wording changes to reflect the collaboration and co-creation processes of sustainable fashion brands. For the PBSF items, items were developed based on relevant conceptual themes from Zhang et al. (2023) and Zou et al. (2024) [33,42]. Two of the items, PBSF1 (environmental benefits) and PBSF2 (social responsibility), were adapted from relevant conceptual insights. The third item, PBSF3 (economic benefits), was developed based on Zhang et al. (2023) [33]. CWASF items were developed based on the Theory of Planned Behavior [37], and were contextually adapted from Bly et al. (2015) and Pandey and Yadav (2023) [55]. PBP items were informed by Liu et al. (2020) [23] and Todeschini et al. (2017) [24], with indicators relating to the reputation, satisfaction, and loyalty of sustainable fashion brand customers.

To assess the measurement validity of the sustainable fashion products purchasing intentions measure, a two-stage pretest procedure was undertaken. Firstly, a small number of items were checked for conceptual coverage, face/content validity, and readability by three domain experts (two academics in the area of sustainable fashion and one industry practitioner). Minor refinements were made in response to their comments. Secondly, a small-scale pilot study was conducted with a sample of 60 participants matched to the target population. While there is no agreed sample size for the pilot study stage, a typical guideline is for a pretest sample of at least 30–50 participants, or 10% of the planned main sample [62]. The pilot study revealed sufficient variation in the measure items with no floor or ceiling effects. All items showed good internal consistency with item–total correlations of >0.30 and overall scale Cronbach’s alpha of >0.70.

### 3.2. Sample and data collection

For establishing the appropriate sample size for SEM, extant literature suggests 200 cases for moderately complex models, a 10:1–20:1 observations to parameters ratio [62,63], or guidelines provided by practitioner-oriented methodologists [64]. For the current study, this translates into a minimum requirement of 240–480 cases, given the four latent constructs and 12 indicators. However, an online sample size calculator available at calculator.net indicates that for a 90% confidence level and a margin of error of 5%, the recommended sample size is 385 cases. In order to obtain sufficient performance-based data, however, the final sample comprises 600 online shoppers.

This study employed quota sampling in four geographic locations in Thailand: Central (Bangkok), North (Chiang Mai), Northeast (Khon Kaen), and South (Phuket). 150 participants were sampled from each region to create overall sample diversity by geographic location, as well as socioeconomics and culture specific to Thailand.

Data collection occurred between 1 November 2024 and 31 January 2025. Participants were aged 18 years and over. The survey was undertaken on a voluntary basis, and participants remained anonymous. Ethical approval was obtained from the Khon Kaen University Ethics Committee for Human Research (KKU-EC: HE673293).

Data were collected via intercept surveys in clothing stores within shopping malls in each region. This mall-based sampling approach may introduce sampling bias, as it tends to overrepresent urban, middle-income consumers while underrepresenting consumers who primarily shop online or support independent sustainable brands. To mitigate this bias, several strategies were employed. First, quota sampling across diverse regions ensured variation in consumer profiles. Second, data source triangulation was applied by collecting responses from multiple retail locations within each region [65]. Third, a structured screening process ensured that only respondents with relevant sustainable fashion experience were included. Additionally, although surveys were conducted in physical retail settings, respondents with both online and offline shopping behaviors were included through screening criteria. These procedures do not eliminate sampling bias; however, they substantially reduce its impact and improve the representativeness and robustness of the sample. While these procedures help reduce sampling bias, the generalizability of the findings is primarily limited to active fashion consumers in urban retail contexts in Thailand. Therefore, caution should be exercised when extending the results to rural populations or consumers who engage exclusively in online or alternative sustainable retail channels.

### 3.3. Data analysis

Given the potential for common method variance (CMV) due to the extensive dataset and use of similar measurement tools, it was necessary to assess CMV before data analysis. CMV, which can arise when variables are measured using the same method and source, particularly with Likert scales, can lead to biased results [66]. Harman’s single-factor test revealed a cumulative variation of 44.49 percent, below the 50 percent threshold, indicating that CMV is not a significant concern in this study. To further strengthen the analysis, a Common Latent Factor (CLF) method was employed to capture any shared variance attributable to the measurement method rather than the constructs. A comparison of standardized factor loadings with and without the CLF revealed a maximum difference of 0.160, comfortably below the 0.20 threshold [67]. This confirms that common method bias does not inflate the observed relationships, providing a more rigorous validation than Harman’s test alone. For data normality, the skewness values in the new dataset range from −0.723 to 0.278, within the acceptable threshold of −1–1, indicating no extreme asymmetry. Similarly, kurtosis values range from −0.303 to 0.566, falling within the acceptable range of −1–1, suggesting no significant deviations from normality in terms of tail weight. Overall, the dataset shows acceptable skewness and kurtosis values, indicating it conforms to normal distribution assumptions.

To analyze these relationships, this study employs covariance-based structural equation modeling (CB-SEM), executed using AMOS software version 26. CB-SEM was selected due to its capability to provide more precise parameter estimates and greater consistency when dealing with large sample sizes, such as those exceeding 250 observations [68]. Unlike partial least squares SEM (PLS-SEM), which is often favored for smaller sample sizes due to its higher statistical power in such contexts, CB-SEM is particularly advantageous for validating complex theoretical models with both structural and measurement components [69]. The use of AMOS allowed for a thorough validation of both the structural model and measurement model, ensuring the robustness of the results and the reliability of the findings.

An exploratory factor analysis (EFA) was performed to examine the underlying structure of the measurement items, particularly for constructs that were partially adapted or newly developed from conceptual and qualitative literature. Following the recommendations of Hair et al. (2019) [64], principal axis factoring with varimax rotation was applied. The measurement model was evaluated using Confirmatory Factor Analysis (CFA) to assess the reliability and validity of the latent constructs. Key assessments included Goodness of Fit, Convergent Validity, and Discriminant Validity. For Goodness of Fit, the model fit was determined using indices such as the Chi-square/df ratio, Comparative Fit Index (CFI), Incremental Fit Index (IFI), Goodness of Fit Index (GFI), and Root Mean Square Error of Approximation (RMSEA) [70]. The acceptable ranges for these indices were defined as follows: a Chi-square/df ratio of less than 3; CFI, IFI, and GFI values greater than 0.90; and an RMSEA value between 0.05 and 0.08. Convergent Validity was ensured by analyzing standardized factor loadings and calculating Composite Reliability (CR) and Cronbach’s alpha for internal consistency [71]. The Average Variance Extracted (AVE) further confirmed that the constructs adequately captured the variance among indicators [72]. Variance Inflation Factor (VIF) was used to identify multi-collinearity among the item variables [64]. Discriminant validity was thoroughly evaluated using both the Fornell and Larcker criterion and the Heterotrait-Monotrait (HTMT) ratio, ensuring that the constructs in the model are empirically distinct. The Fornell and Larcker method compares the square root of AVE with the correlations between constructs, while the HTMT ratio assesses the strength of correlations between constructs to identify potential overlaps [73]. Following this, structural models were analyzed to explore the relationships between constructs, evaluating path coefficients and model fit using relevant indices.

Bootstrapping was employed to examine mediation effects, a method that provides reliable estimates of indirect effects by repeatedly resampling the data. This approach offers bias-corrected confidence intervals, allowing a more accurate interpretation of mediation effects [74]. Bootstrapping is particularly advantageous in mediation analysis as it avoids the assumption of normality in the distribution of indirect effects, thus providing more robust and reliable results.

## 4. Results

A total of 617 responses were obtained, and after data cleansing, 602 valid responses were retained, resulting in a response rate of approximately 97.6%. According to Table 1, the demographic data reveal that the majority of respondents are female (62.3%) and primarily fall within the 30−39 age group (37.7%). A significant portion of the sample holds a Bachelor’s degree (48.5%), with most respondents earning between 840−1,680 USD monthly (35.2%). In terms of shopping behavior, a large percentage of respondents prefer online shopping (42.9%), while others are evenly split between in-store shopping (26.9%) and a mix of both (30.2%).

**Table 1:** Demographic profiles.

Demographics	Measure	Frequency (n)	Percentage (%)
Gender	Male	190	31.60%
	Female	375	62.30%
	Prefer not to say	37	6.10%
Age (Year)	20-29	143	23.80%
	30-39	227	37.70%
	40-49	157	26.10%
	50 and above	75	12.50%
Education	Below high school	42	7.00%
	High school diploma or equivalent	165	27.40%
	Bachelor’s degree	292	48.50%
	Graduate degree	103	17.10%
Monthly Income (USD)	Less than 840	97	16.10%
	840−1,680	165	27.40%
	840−1,680	212	35.20%
	2,800 and above	128	21.30%
Shopping Behavior	Primarily online	258	42.90%
	Primarily in-store	162	26.90%
	A mix of both	182	30.20%

*Note: Conversion based on an exchange rate of approximately 1 THB = 0.028 USD (the average as of September 2025)

### 4.1. Exploratory Factor Analysis (EFA)

Before performing factor extraction, it was crucial to establish whether the data were suitable for performing a factor analysis. The KMO measure was 0.912, which is classified as excellent, thereby pointing towards very good sampling adequacy. Additionally, Bartlett’s test of sphericity was found to be significant (χ² = 7241.55, p < 0.001), which indicates that factor analysis was appropriate to use. After conducting an exploratory factor analysis using principal component analysis with Varimax rotation, a clear four-factor solution emerged. As can be seen from Table 2, all the items loaded strongly onto their respective factors (with most having loadings above 0.70). None of the items were found to have problematic cross-loadings. All the items had high communality values, with the lowest being 0.638 and the highest 0.918, thus demonstrating high levels of explained variance. Some of the lower loadings have been suppressed in order to increase the readability of the table. The results also show the amount of variance each of the retained components accounted for, as well as the component-to-component correlations. The four factors explained a total of 78.14% of the variance; with the largest contribution being Factor 1, Perceived Collaborative Innovation (PCI) which explained 42.15%, followed by Factor 2, Perceived Benefits of Sustainable Fashion (PBSF) which explained 15.33%, then Factor 3, Consumer Willingness to Adopt Sustainable Fashion (CWASF) accounting for 11.22% of variance, and finally Factor 4, Perceived Brand Performance (PBP) accounting for 9.44%.

**Table 2:** Comprehensive exploratory factor analysis results.

Factor Loadings (Varimax Rotation)					
Item	Factor 1 (PCI)	Factor 2 (PBSF)	Factor 3 (CWASF)	Factor 4 (PBP)	Communality
PCI1	0.951	—	—	—	0.904
PCI2	0.958	—	—	—	0.918
PCI3	0.861	—	—	—	0.741
PBSF1	—	0.911	—	—	0.83
PBSF2	—	0.912	—	—	0.832
PBSF3	—	0.847	—	—	0.717
CWASF1	—	—	0.835	—	0.697
CWASF2	—	—	0.904	—	0.817
CWASF3	—	—	0.799	—	0.638
PBP1	—	—	—	0.817	0.667
PBP2	—	—	—	0.855	0.731
PBP3	—	—	—	0.938	0.88
Factor Statistics & Correlation Matrix					
Component	PCI	PBSF	CWASF	PBP	Definition
Factor 1 (PCI)	1	0.612	0.545	0.49	Perceived Collaborative Innovation
Factor 2 (PBSF)	0.612	1	0.68	0.525	Perceived Benefits of Sustainable Fashion
Factor 3 (CWASF)	0.545	0.68	1	0.595	Consumer Willingness to Adopt Sustainable Fashion
Factor 4 (PBP)	0.49	0.525	0.595	1	Perceived Brand Performance
Eigenvalue	5.058	1.84	1.346	1.133	
% Variance	42.15%	15.33%	11.22%	9.44%	
Cumulative %	42.15%	57.49%	68.71%	78.15%	

### 4.2. Measurement model

The goodness of fit (GOF) indices for the measurement model indicate a strong fit between the model and the observed data. Specifically, the CFI of 0.987 exceeds the commonly accepted threshold of 0.95, suggesting an excellent fit. The GFI at 0.967 also surpasses the threshold of 0.90, further confirming the model’s adequacy. Similarly, the IFI of 0.987, which is above the 0.95 benchmark, indicates that the model fits well compared to a null model. The RMSEA is 0.056, which falls within the acceptable range of 0.05 to 0.08, indicating a reasonable error of approximation. Lastly, the Chi-square/df ratio of 2.864 is below the threshold of 3.0, which suggests that the model is an acceptable fit for the data. Collectively, these indices demonstrate that the measurement model has achieved an excellent fit, validating the robustness of the constructs under investigation.

Table 3 focuses on the convergent validity of the measurement model by evaluating key constructs: PCI, PBSF, CWASF, and PBP. Each construct’s indicators (PCI1–PCI3, PBSF1–PBSF3, CWASF1–CWASF3, and PBP1–PBP3) exhibit high factor loadings, all significant at the p < 0.001 level, confirming strong relationships among the indicators for each construct. Cronbach’s alpha values, ranging from 0.887 to 0.945, exceed the 0.70 threshold, indicating excellent internal consistency. The AVE values, ranging from 0.718 to 0.856, are well above the 0.50 threshold, demonstrating that a significant portion of the variance is captured by the constructs. Additionally, CR values, ranging from 0.884 to 0.942, surpass the 0.70 threshold, further validating the reliability and accuracy of the measurement model. Furthermore, multicollinearity among indicators was assessed using the variance inflation factor (VIF). The VIF values range from 1.64 to 4.45, which are below the commonly accepted threshold of 5.0 [64], indicating that multicollinearity is not a serious concern in the measurement model. These findings collectively affirm the strong convergent validity of the constructs within the study.

**Table 3:** Convergent Validity.

Construct	Indicator	Estimate (>0.70)	p-Value	VIF (<5.0)	Cronbach’s Alpha (>0.70)	AVE (>0.50)	CR (>0.70)
Perceived Collaborative Innovation	PCI1	0.951	***	4.21	0.945	0.856	0.942
	PCI2	0.958	***	4.45			
	PCI3	0.861	***	2.12			
Perceived Benefits of Sustainable Fashion	PBSF1	0.911	***	3.05	0.925	0.793	0.920
	PBSF2	0.912	***	3.10			
	PBSF3	0.847	***	1.98			
Consumer Willingness to Adopt Sustainable Fashion	CWASF1	0.835	***	1.85	0.887	0.718	0.884
	CWASF2	0.904	***	2.62			
	CWASF3	0.799	***	1.64			
Perceived Brand Performance	PBP1	0.817	***	1.75	0.930	0.759	0.904
	PBP2	0.855	***	2.08			
	PBP3	0.938	***	3.85			

Note: *** denotes significance p-value < 0.001

Table 4 provides a thorough assessment of discriminant validity by presenting three types of values in distinct sections: diagonal, lower off-diagonal triangle, and upper off-diagonal triangle. The diagonal elements, such as 0.925 for PCI, 0.891 for PBSF, 0.847 for CWASF, and 0.871 for PBP, represent the square root of the AVE for each construct, with these values emphasized in italics. According to the Fornell-Larcker criterion, these diagonal values should be higher than the corresponding correlations between the constructs, which are displayed in the lower off-diagonal triangle. This comparison ensures that each construct is more strongly related to its own indicators than to those of other constructs, confirming discriminant validity. The results show that all diagonal values exceed the inter-construct correlations, satisfying the Fornell–Larcker criterion. Additionally, the upper off-diagonal triangle contains the HTMT ratios, which further test discriminant validity. HTMT values below the threshold of 0.85 indicate that the constructs are sufficiently distinct from one another. All HTMT values fall below this threshold, indicating that discriminant validity is established. This combined evidence from the Fornell-Larcker criterion and HTMT ratios confirms that the constructs are differentiated, supporting the reliability and validity of the measurement model.

**Table 4:** Discriminant validity – fornel and larcker criterion vs HTMT Ratios.

Construct	PCI	PBSF	CWASF	PBP
PCI	*0.925*	0.654	0.595	0.522
PBSF	0.612	*0.891*	0.751	0.566
CWASF	0.545	0.68	*0.847*	0.655
PBP	0.49	0.525	0.595	*0.871*

Note: Values in italics represent the square root of the Average Variance Extracted (AVE).

### 4.3. Structural model

The structural model analysis confirms the hypothesized relationships, with all paths being statistically significant. The GOF indices demonstrate a robust model fit: CFI = 0.987 (threshold > 0.90), GFI = 0.967 (threshold > 0.90), IFI = 0.987 (threshold > 0.90), and RMSEA = 0.056 (threshold < 0.08). Additionally, the Chi-square/df ratio is 2.864, which is within the acceptable range (threshold < 3). These indices provide a strong basis for interpreting the path coefficients and affirm the model’s overall validity. Table 5 summarizes the hypothesis test results, with standardized coefficients and p-values reported for direct effects, and bootstrapped confidence intervals provided for mediation (indirect) effects.

**Table 5:** Hypothesis test results – direct and indirect effects.

Effect Type	Hypothesis	Path	Standardized Coefficient (β)	Lower Bound	Upper Bound	P-value	R² (Dependent Variable)	Decision
Direct Effect	H1	PCI → PBSF	0.265	–	–	0.001*	R² (PBSF) = 0.21	Supported
	H2	PCI → CWASF	0.193	–	–	0.001*	–	Supported
	H3	PCI → PBP	0.198	–	–	0.001*	–	Supported
	H4	PBSF → CWASF	0.351	–	–	0.001*	R² (CWASF) = 0.42	Supported
	H5	CWASF → PBP	0.351	–	–	0.002*	R² (PBP) = 0.47	Supported
Indirect Effect	H6	PCI → PBSF → CWASF	0.093	0.042	0.15	0.001*	–	Supported (Partial Mediation)
	H7	PCI → CWASF → PBP	0.068	0.042	0.105	0.001*	–	Supported (Partial Mediation)
	H8	PCI → PBSF → CWASF → PBP	0.035	0.018	0.065	0.001*	–	Supported (Partial Mediation)
Total Effect	–	PCI → PBSF	0.265	0.136	0.391	0.001*	–	–
	–	PCI → CWASF	0.286	0.166	0.390	0.001*	–	–
	–	PCI → PBP	0.301	0.217	0.403	0.001*	–	–

Note: * denotes significance at p < 0.01. R² values represent variance explained in the endogenous constructs. Mediation decisions are based on bootstrapped indirect effect tests (5,000 resamples, bias-corrected).

Results of the structural model analysis provided support for several significant direct effects. First, results indicated a significant path from PCI to PBSF, standardized path coefficient = 0.265, t-value = 2.58, p = 0.001, supporting H1. Second, findings supported H2, indicating a significant path from PCI to CWASF, standardized path coefficient = 0.193, t-value = 2.06, p = 0.001. In addition, the finding that PCI had a significant positive effect on PBP, standardized path coefficient = 0.198, t-value = 2.08, p = 0.001, supported H3. Two other significant paths were also observed. Specifically, the finding that PBSFBS had a significant positive effect on CWASF, standardized path coefficient = 0.351, t-value = 3.09, p = 0.001, supported H4. In addition, the finding that CWASF had a significant positive effect on PBP, standardized path coefficient = 0.351, t-value = 2.99, p = 0.002, supported H5. Following the rules of thumb by Cohen (1988), which indicate that |β| between 0.10 and 0.29 is small, |β| between 0.30 and 0.49 is medium, and |β| ≥ 0.50 is large, these direct effects are characterized by small to medium effect sizes. Specifically, the direct effects of PCI on PBSF and PBP are small. However, the consumer-related effects (PBSF and CWASF) have somewhat stronger effects on adoption (PBP) and on FBS-relevant outcomes (CWASF).

The indirect effects suggest some interesting new mediation paths between PCI and CWASF and/or PBP. For H6, our results indicate that PCI has an indirect effect on CWASF through PBSF, such that when consumers perceive stronger PCI benefits, they are more willing to adopt sustainable fashion. The estimated indirect effect for H6 is 0.093 (95% CI: 0.042, 0.150, p-value = 0.001). For H7, PCI has an indirect effect on PBP through CWASF, such that when consumers are more willing to adopt sustainable fashion, they are more likely to perceive positive PBP. The estimated indirect effect for H7 is 0.068 (95% CI: 0.042, 0.105, p-value = 0.001). Lastly, for H8, we find that PCI positively affects PBP through both steps. PCI first leads to increased perceived benefits (PBSF) that, in turn, increases willingness to adopt (CWASF), and then CWASF leads to increased PBP. The estimated indirect effect of PCI on PBP through PBSF and CWASF is 0.035 (95% CI: 0.018, 0.065, p-value = 0.001). It is noteworthy that these indirect effects are somewhat smaller in magnitude than the direct effects reported above, but each of the indirect effects has substantive significance. Overall, the results demonstrate the crucial role that consumer perceptions of PCI play in the collaboratively innovative fashion company’s ability to perform better in the market.

The total effects, combining both direct and indirect effects, provide a more complete understanding of how PCI is related to PBSF, CWASF, and PBP. The total effect of PCI on PBSF is 0.265 (p = 0.001). This is the same as the direct effect since there are no other mediating paths. The total effect of PCI on CWASF is 0.286 (p = 0.001). This is the sum of the effect of PCI on CWASF and the effect of PCI on PBSF multiplied by the effect of PBSF on CWASF: 0.193 + 0.093 = 0.286. Finally, the overall total effect of PCI on PBP is 0.301 (p = 0.001). This is the sum of the direct effect PCI to PBP (0.198) and the indirect effect of PCI on PBP through CWASF (0.068) and the indirect effect of PCI on PBP through both PBSF and CWASF (0.035). Thus, collaborative innovation has a significant impact on PBP through the engagement of consumer-focused factors. Significantly, all of the mediation paths identified above were significant. This is distinct from partial mediation, wherein the direct path remains significant along with the indirect path. In partial mediation, both the direct and indirect effects are significant and together explain the relationship between two variables. In this study, the direct effects from PCI to CWASF and from CWASF to PBP remained significant. Hence, the findings are one of partial mediation. Importantly, the total effect for PCI on PBP (0.301) is seen as medium effect.

In addition to the estimated path coefficients, it is also of interest to have an indication of the model’s explanatory power regarding the endogenous constructs. As can be observed from the Table 8, PCI explains 21% of the variance in PBSF (R² = 0.21). When both PCI and PBSF are included in the equation for CWASF, the combination of both variables explain 42% of the variance in CWASF (R² = 0.42). Moreover, the three variables together explain 47% of the variance in PBP (R² = 0.47). Hair et al. (2019) [64] propose the following guidelines for R² values obtained in SEM studies: R² values close to 0.20 indicate weak explanatory power, values around 0.30 indicate moderate explanatory power, and values of 0.40 or more indicate strong explanatory power. Thus, the model seems to explain moderately PBSF and strongly CWASF and PBP.

**Fig 2:**
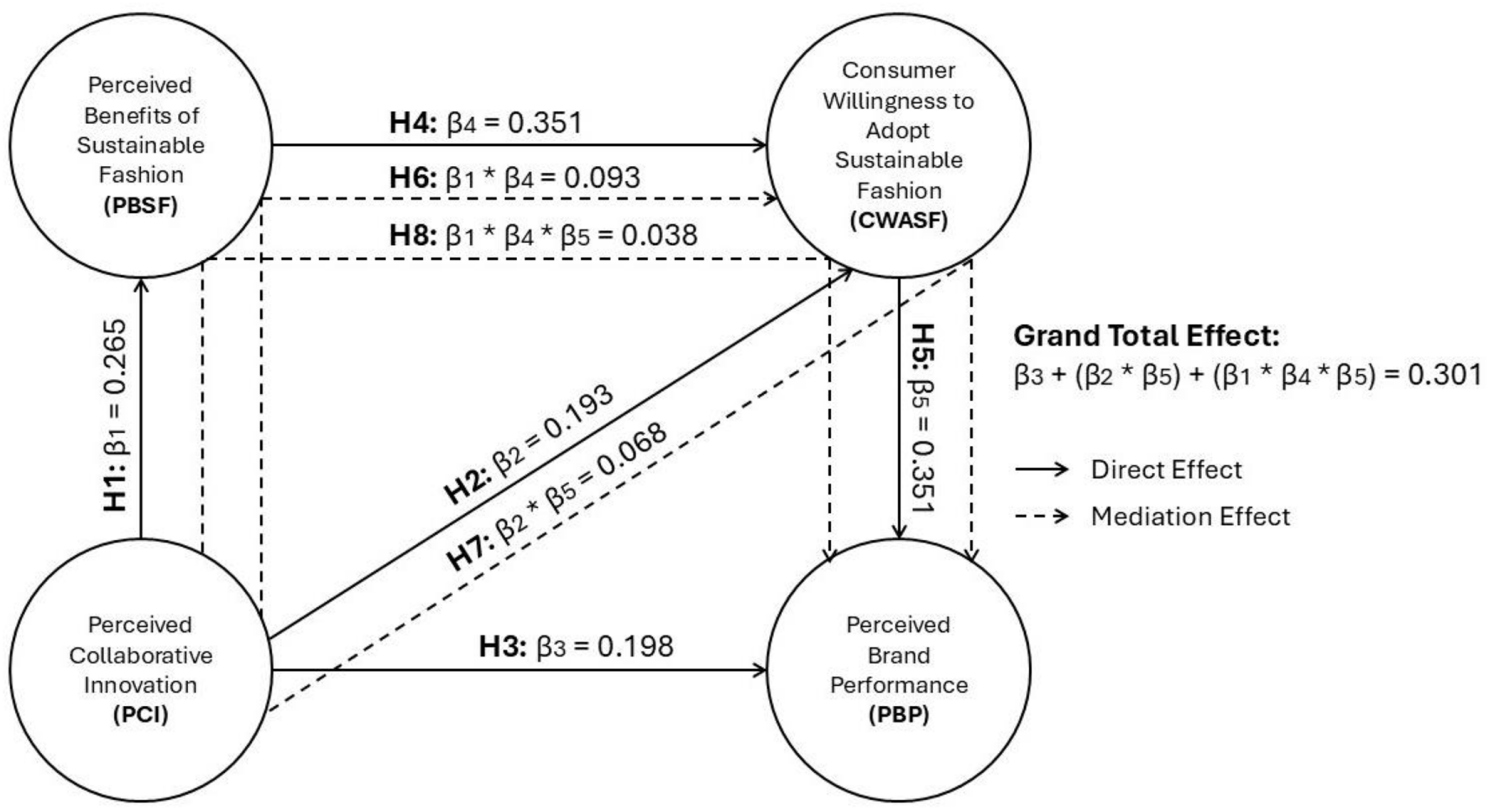
Complete structural model – direct effects, mediation effects, and grand total effect.

Fig 2 Presentation of the complete structural model for direct effects, mediation effects and total effects. Detailed information about Fig 2 is provided in the discussion section.

## 5. Discussion

The SEM analysis provides valuable insights into the relationships among PCI, PBSF, CWASF, and PBP within the sustainable fashion industry. By rigorously examining both direct and indirect effects, this section robustly connects empirical findings to existing literature and theoretical frameworks, offering a comprehensive understanding of how innovation and consumer engagement contribute to market success. In particular, the findings provide further support for Open Innovation Theory [8], Diffusion of Innovations theory [51], and perceived value framework, all of which help explain the mechanisms by which consumer perceptions and adoption behaviors link collaborative innovation with brand performance.

### 5.1. Direct effects

Results from the structural model analysis support several significant direct effects from the exogenous variables (PCI) to the endogenous construct (PBSF). These findings support the theoretical hypotheses put forth in the paper. Firstly, the direct effect of PCI on PBSF (β = 0.265) supports H1. Positive collaborative innovation within fashion companies leads to a higher PBSF among consumers, which in turn will have products that are in line with the values of these consumers. This finding supports Open Innovation Theory, as seen in Chesbrough (2003) [8], which states that the inclusion of ideas and knowledge from outside the company can generate innovations for fashion companies, with a view to increasing the value of products for consumers (from leather goods to garments, footwear, accessories, and so on). This also corroborates Zeithaml (1988) [35], the value framework, which identifies the criteria on the basis of which consumers will evaluate sustainable innovations, particularly if they are fair in terms of environmental and social benefits. In general, sustainable innovations are perceived by consumers as having more value if they are consistent with the perceived benefits and decreased costs. From a practical point of view, fashion companies can start collaborations such as co-design projects with consumers, partnerships with sustainable raw materials producers (textiles, for example), and social and environmentally friendly partnerships with NGOs that provide credible certifications to indicate fair labor practices. The benefits of sustainability will be made visible to consumers, making them more likely to perceive higher environmental and social value.

This direct effect from PCI to CWASF also supports H2 and reveals that PCI has a significant and positive effect on CWASF. By collaborating and innovating, fashion companies are better able to introduce products that meet the expectations of consumers concerning sustainability [75]. Therefore, as per the argument of Chesbrough (2003) [8], collaborative innovation is more likely to create products that consumers are willing to adopt. Additionally, the Diffusion of Innovations theory by Rogers (2003) [51] identifies relative advantage, compatibility, and observability as drivers of adoption. Collaborative innovation improves these dimensions when translated into consumer-relevant outcomes, or CF.

The relationship between PCI and PBP was found to be positive and significant with a coefficient of 0.198 (p = 0.001), thereby supporting H3. Industry leaders rely upon PCI to achieve brand performance. The connection between PCI and brand performance is real and runs through consumer adoption of innovation, whereby innovations created and pushed by industry leaders translate into increased consumer engagement that, in turn, translates into market success for the firm. Well established in the literature, innovation is found to have a positive effect on a firm’s market success [44]. Furthermore, innovation explains how firms create value for their brands by meeting the demands of increasingly sophisticated customers and by differentiating themselves from rivals [9]. By drawing on the diffusion of innovations model, increased adoption of innovations by consumers leads to increased legitimacy and hence a stronger reputational-based brand performance.

In addition to the indirect effect of PBSF through CWASF, there exists a positive direct effect of PBSF on CWASF (β = 0.351). This also supports H4 and further validates the role of PBSF in sustainable fashion marketing [55]. Consumer willingness to adopt sustainable fashion is primarily driven by consumer perceived benefits of sustainable fashion (CWASF). Perceived environmental, social, and economic benefits are among the main drivers for sustainable consumption. As suggested by Zeithaml (1988) [35], perceived value is a key antecedent to customer behavior in terms of purchasing a product when perceived benefits are greater than perceived costs. As such, the perceived benefits of sustainable fashion, such as wearing organic cotton products with a reduced carbon footprint, sustainable fashion with fair trade practices, sustainable fashion that is durable guarantee, etc., can make sustainable fashion more attractive to adopt by consumers.

Finally, the direct effect of CWASF on PBP (β = 0.351) supports H5. CWASF positively affects PBP, which in turn improves brand performance. Sustainability has become an increasingly important consideration for brands, especially as the market moves towards greater environmental sustainability [76]. This is because non-financial brand performance measures, such as consumer loyalty, brand image, and overall satisfaction with a brand’s environmental and social credentials, are highly influential [77]. As Rogers (2003) [51] notes, the adoption of innovations by potential users not only benefits the individual adopter but also can provide legitimacy and visibility for the brand that has introduced the innovation. As such, the findings support the argument that consumer behaviour affects brand performance through sustainability values. This effect is particularly relevant in markets where consumer values and perceptions are critical [77]. Practically speaking, consumers’ willingness to pay a premium for sustainable products and services can translate into brand loyalty when the brand effectively communicates its sustainability credentials, engages with them post-purchase (e.g., recycling initiatives), and delivers on the promises made around its brand’s ethical position. By doing so, a brand can reap long-term brand performance benefits.

### 5.2. Indirect effects

An examination of indirect effects reveals important links between PCI and both CWASF and PBP via consumer perception and engagement.

This indirect effect of PCI on CWASF through PBSF (β = 0.093) reveals that the positive effect of PCI on CWASF is significantly enhanced by PBSF. This is because in order to translate PCI into consumer behavior or intention, consumers’ perceptions of environmental, social and economic benefits in sustainable fashion play important roles. As innovations perceived as beneficial tend to have greater impact on consumers’ attitudes and behavior than those perceived as neutral or detrimental, PBSF acts as an important mediator to translate PCI, the capability to create innovative products and services, into consumer behavior or intention. This highlights the importance of enabling consumers to perceive the advantages of sustainable fashion. In this sense, simply having PCI capabilities is not enough for these innovations to have an effect on CWASF; they must be effectively communicated to consumers. This process follows the value concept framework by Zeithaml (1988) [35], where perceived value acts as a mediator of the effect of innovation on adoption intentions.

The indirect effect of PCI on PBP through CWASF (β = 0.068) is noteworthy in view of the intermediary role of CWASF influenced by PCI. From this perspective, consumer engagement with brands, far from being a passive by-product of innovations, plays a critical role in their success. The large body of research that finds that consumer behavior has a profound effect on brand performance is therefore borne out in this study, especially in a market driven by consumer values and perceptions [77]. Consequently, companies should strive to create customer workflows that foster CWASF to boost PBP, which encompasses not only financial performance but also important non-financial performance indicators such as customer satisfaction and brand loyalty. From the perspective of Rogers (2003) [51], the willingness of consumers to adopt innovations legitimates and fuels their impact on brand performance.

Moreover, the indirect effect from PCI to PBP through the sequential pathway PCI → PBSF → CWASF → PBP (β = 0.035) further shows that consumer engagement is essential for realizing the benefits of PCI for sustainable fashion brand performance. This effect indicates that while mere innovation is important, both direct and indirect contributions are necessary for brand performance. Direct contributions come from novel products and services created by collaborative business models while their full impact is realized when consumers perceive such innovations as beneficial [12] and are willing to adopt [8,9]. In this sense, the sequential mediation further supports the use of Zeithaml’s (1988) [35] perceived value theory and Rogers’ (2003) [51] adoption theory. Specifically, the two constructs of perceived benefits for and willingness to adopt sustainable fashion innovations serve as cognitive and behavioral processes linking PCI to sustainable brand performance.

### 5.3. Total effects and sequential mediation analysis

**Fig 3:**
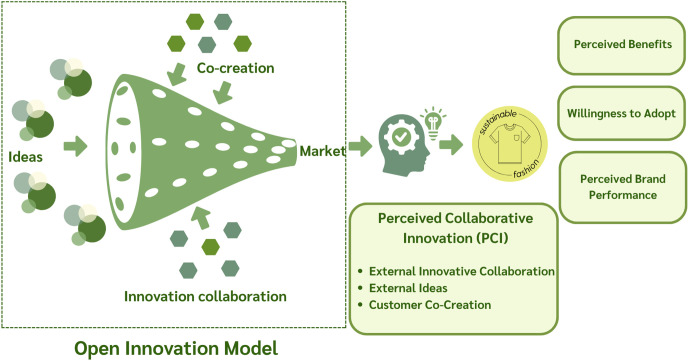
Extended open innovation framework for sustainable fashion.

The total effects analysis clarifies how PCI shapes both CWASF and PBP, highlighting the interconnected role of consumer engagement in translating collaborative innovation into brand outcomes. Fig 3 explains the mechanism of the extended open innovation framework for sustainable fashion, focusing on collaborative innovation. Fig 3 illustrates the mechanism of the extended open innovation framework for sustainable fashion, emphasizing collaborative innovation and its effects on consumer responses. The framework comprises two key dimensions: the Open Innovation Dimension and the Consumer Response Dimension. The Open Innovation Dimension is represented by Perceived Collaborative Innovation (PCI), reflecting how firms leverage external collaboration and co-creation to develop sustainable fashion offerings. The Consumer Response Dimension includes Perceived Benefits (PB), Willingness to Adopt (CWASF), and Perceived Brand Performance (PBP), capturing cognitive evaluation, behavioral intention, and brand outcomes, respectively. The directional arrows indicate both direct and indirect relationships. Specifically, PCI influences PB and CWASF, which in turn affect PBP. The sequential pathway from PB to CWASF highlights the cognitive–behavioral mechanism through which collaborative innovation translates into consumer adoption and brand performance.

Overall, the framework extends traditional open innovation models by explicitly linking firm-level innovation with consumer perceptions and behavioral outcomes.The direct effect of PCI on CWASF (β = 0.193) shows that innovation significantly influences consumers’ willingness to adopt sustainable fashion, confirming that collaborative practices strongly shape sustainable purchasing decisions [8,9]. Beyond this direct path, the indirect effect of PCI on CWASF through PBSF (β = 0.093) emphasizes that consumers must perceive clear environmental, social, and economic benefits before innovation translates into adoption. This pathway aligns with Zeithaml’s (1988) [35] perceived value framework, in which adoption intentions are strengthened when perceived benefits outweigh costs, and with Rogers’ (2003) [51] Diffusion of Innovations theory, which highlights relative advantage, compatibility, and observability as key drivers of adoption. Taken together, the total effect of PCI on CWASF (β = 0.286) illustrates that consumer willingness is maximized when both collaborative innovation and the communication of its benefits are present, reinforcing prior findings on the role of consumer-centered communication in sustainable fashion [75,78]. From a theoretical perspective, this pathway reflects the Open Innovation Dimension, as firms leverage external collaborations to co-create sustainable solutions, while PBSF and CWASF reflect the Consumer Engagement Dimension by translating these innovations into perceived value and adoption behaviors at the consumer level.

PCI also has both direct and indirect effects on PBP. Firstly, the direct effect of PCI on PBP (β = 0.198) indicates that innovation capability is valuable for enhancing non-financial brand performance, including loyalty and satisfaction [44,48]. There are also additional indirect effects, where consumer willingness to adopt innovation acts as a significant mediator, firstly through CWASF (β = 0.068), and secondly through a sequential pathway for PBSF and CWASF (β = 0.035). The total effect of PCI on PBP is β = 0.301, indicating that the highest brand performance is obtained by integrating innovation with consumer perceptions and their willingness to adopt innovation [23,55].

However, collaborative innovation may not result in improvements to PBP in all circumstances. Excessive openness can lead to increased coordination costs, knowledge leakage, and decreased brand identity [27,20]. Chesbrough (2003) [8] also highlighted that the key to successful open innovation is the appropriate and strategic management of external knowledge to ensure that it translates into improved PBP. Furthermore, while PCI contains positive consumer effects, there are also negative consumer reactions to sustainability claims, such as greenwashing and self-deception [79]. Thus, PCI has the potential to foster ambivalence and even negative attitudes, which would undermine trust in brands that employ collaborative innovation strategies.

In addition to contributing positively to brand performance in a direct way, PCI also indirectly enhances brand performance by means of consumer engagement with collaborative innovation. The findings of this study, furthermore, suggest that it is only by pairing PCI with such engagement mechanisms that the full potential of PCI can be realised. It is through the psychological processes underlying consumer awareness of PBSF (perceived benefits of sustainable fashion) and behavioural change towards CWASF (consumers willing to adopt sustainable fashion) that collaborative business-level innovation can translate into brand performance. Hence, this study finds that it is necessary to integrate the Open Innovation Dimension with the Consumer Engagement Dimension for sustainable firm-level collaboration to translate into sustainable competitive advantage. By applying and extending existing innovation and engagement theory, this study finds that successful long-term brand performance in the sustainable fashion industry is driven by PCI, perceived value, and adoption.

## 6. Implications

This section explores the strategic implications for industry practice, theoretical contributions to existing knowledge, and an overview of limitations and directions for future research. These discussions deepen our understanding of the role of collaborative innovation and consumer engagement in the sustainable fashion industry, offering both practical and academic insights.

### 6.1. Practical and managerial implications

The findings of this study have important practical implications for sustainable fashion brands that seek to translate collaborative fashion innovation into consumer engagement and brand performance. While results show that perceived collaborative innovation (PCI) has a direct and indirect effect on consumer willingness to engage with sustainable fashion and brand performance, it is most effective when it is translated into perceived consumer value by firms and stimulates consumer behaviour. In practice, collaborative fashion innovation needs to be translated into concrete actions by firms and effectively implemented towards consumers. Therefore, mere adoption of collaborative innovation is not sufficient; managers must operationalise collaborative innovation.

Firms first need to make the perceived benefits of sustainable fashion (PBSF) operational by converting their collaborative innovation into perceived value for their consumers. Sustainability in products and processes must be made perceptible in terms of environmental, social, and economic added value. Eco-labels, QR codes, or increased supply chain transparency can serve as ways to make the added value visible to consumers and to make sustainable claims verifiable. In Thailand, collaboration with local crafts can be a means to leverage sustainable practices within culturally relevant innovation and create strong perceived authenticity and emotional connections.

Next, firms should maximise consumer willingness to adopt sustainable fashion (CWASF) by means of effective engagement. The findings above highlight the pivotal role of consumer willingness to translate perceived sustainable fashion benefits into brand performance. To this end, brands are urged to capitalise on digital platforms, social media, as well as influencer and interactive marketing measures in order to normalise sustainable consumption. For instance, partnerships with environmentally active influencers, user-generated content campaigns (UGC); virtual and live-streaming shopping experiences can facilitate engagement. Accessible pricing strategies, basic sustainable products, and easy product comparisons can additionally remove adoption barriers and make sustainable fashion desirable and relevant. There are many examples of brands putting these approaches into practice. For example, Thai fashion e-retailer, Pomelo Fashion ran a campaign in Thailand promoting their sustainable collections. As stated in the literature, consumer willingness to adopt sustainable fashion is driven by perceived benefits and social influence [55,77].

Third, firms must develop the indirect effects of PBSF and CWASF by incorporating innovation, communication, and customer experience (PCI) into their sustainability strategies. Taking a long-term perspective, the model suggests that the effectiveness of collaborative innovation is contingent upon how firms are able to connect sustainability benefits with consumer behavior. Firms that engage consumers through integrated campaigns and communicate how their purchases reduce environmental harm or support fair work practices can increase perceived value and reinforce commitment to purchasing sustainability features (PBP). For instance, a firm can link a sustainability feature to rewards points or promote a recyclable or reusable product packaging through a trade-in incentive, thereby reinforcing brand loyalty and long-term consumer experience.

Fourth, businesses should build on the direct impact of collaborative innovation by strengthening their innovation capabilities and partnerships. This could involve co-creation with consumers, collaboration with sustainable suppliers, or partnerships with local communities to develop products that are aligned with sustainability values. Technologies, such as blockchain, are beginning to create new business models that leverage such tools to create transparent and trustworthy production, distribution, and consumption systems, while increasing the quality and efficiency of the production and operational processes, and enhancing brand reputation [8,21].

Managers also need to consider the practical implementation difficulties related to collaborative innovation. Large investments are required in terms of technology as well as in building up contacts and network relations with customers, suppliers, and other stakeholders in the value chain. Achieving complete transparency throughout the supply chain is also a major challenge. For the smaller firm, the main constraint will be that of resources. While larger rivals can spread their costs over a greater volume of sales and introduce one new sustainable product or service after another, the smaller firm will be unable to follow this approach. Indeed, it is only by starting small that managers can avoid taking on unnecessary risk and gradually build up capabilities and move towards more complex collaborative forms of innovation. As Wang et al. (2024) [9] point out, smaller firms require a step-by-step approach to developing their innovation capabilities. They may begin by establishing a few partnerships on a small scale and then gradually extend their involvement in collaborative innovation, whether through technology or new markets for existing products. Chesbrough (2003) [8] refers to such an evolutionary process as ‘open innovation’.

This study asserts that collaborative innovation should not be viewed as a discrete process, but rather as one component of a larger, consumer-focused methodology for growth that coordinates innovation activities, value communication, and behavioral engagement mechanisms. Successful implementation of this consumer-focused approach can help companies increase consumer adoption, drive brand performance, and deliver sustained competitive advantage in the increasingly dynamic sustainable fashion market.

### 6.2. Theoretical contributions

This paper provides a range of theoretical contributions to our understanding of consumer behavior within the sustainable fashion industry.

Extending Open Innovation Theory to a Consumer-Centric Model: While Open Innovation Theory speaks to the flow of knowledge internally and externally across a company for product development and operational efficiency [8]. The specific lens of collaborative innovation focuses on the co-creation of solutions with stakeholders, particularly within a more consumer-centric framework. Collaborative innovation specifically draws on the involvement of customers in addressing challenges for co-creating solutions [28]. Not all open innovation initiatives are as focused on customers as the collaborative innovation approach takes, with firm-centric open innovation initiatives instead involving supply chain partners, universities, and other industry alliances. The current study extends this framework by employing a more consumer-centric perspective, demonstrating the critical role of consumers and their engagement through platforms such as PBSF and CWASF to translate collaborative innovation initiatives into actual outcomes. Collaborative innovation outcomes are seen to extend beyond mere technical innovation to encompass innovation that is highly congruent with the values and expectations of consumers [9]. Drawing on Rogers’ (2003) [51] adoption perspective and value [Zeithaml, 1988] [35], the key drivers of sustainable fashion innovation adoption from the consumer’s perspective are perceived benefits and compatibility. The study thereby integrates a consumer behavior perspective into our understanding of collaborative innovation.

Enhancing Understanding of Sustainable Fashion Dynamics: An Open and Collaborative Approach—A Typology Development: This study presents empirical findings based on the application of Open Innovation Theory to the sustainable fashion industry. To date, while sustainable fashion has been discussed largely separately from other innovation practices [75,78], in this study, the findings demonstrate that sustainable PCI through the application of PBSF (via CWASF) and PBP creates a virtuous sustainable PCI dynamics. PCI operates through collaborative practices with consumers to generate a positive virtuous circle, driving desirable consumer engagement. By fostering collaborative approaches, firms can enhance engagement of their consumers (those directly interacting with the firm), creating a positive reinforcing cycle or feedback loop that not only increases brand performance through consumer perceptions and willingness to pass on sustainable fashion recommendations and actions (sustainability values) but also via sales, market share, and customer loyalty that is critical to sustain innovation and consumer commitment. Importantly, firm-level collaborative practices (Open Innovation Dimension) must be linked with consumer-level evaluations and behavior (Consumer Engagement Dimension) in order to be effective.

Empirical Validation of Consumer Engagement as a Mediator: This study empirically tests and validates the proposition that consumer engagement acts as a mediator between collaborative innovation and brand performance. The findings from the partial and mediation analyses demonstrate that positive brand-focused sustainable fashion features (PBSF) and customer word-of-mouth about sustainable fashion attributes (CWASF) act as significant mediators between positive perceptions of collaborative innovation (PCI) and positive brand performance (PBP). Specifically, parallel and sequential mediation are observed, thereby confirming H1 (PBSF mediates the effect of PCI on PBP) and supporting H2 (CWASF mediates the effect of PCI on PBP), as well as establishing the additional indirect effects (PCI → PBSF → CWASF → PBP), in support of H8. Importantly, the indirect effects of PCI through engagement channels add incremental explanatory value to the prediction of PBP, thus underscoring the empirical significance of collaborating with consumers to drive brand performance by communicating innovation’s value Propositions [16,77]. In advancing the theoretical framework, the study distinguishes cognitive engagement (PBSF) from behavioral engagement (CWASF) and highlights the distinct contributions of engagement with sustainable fashion in differentiating sustainable from conventional fashion brands. The findings have practical implications for sustainable fashion brands seeking to leverage innovative approaches to engagement and to drive brand performance by valuing consumers as sources of innovative ideas, values, and commitment to sustainability that can be harnessed through consumer-centered innovation strategies.

### 6.3. Limitations and future research

This study illuminated collaborative innovation and consumer behavior for sustainable fashion; however, some limitations must be acknowledged. The study focused on a Thai sample, and as such, the findings might not be generalizable to other cultures or markets. In addition, the study measured brand performance solely from the consumer’s perspective, and including the perspectives of businesses and industry experts could have provided a richer, more holistic measure of brand performance across the various perspectives. Even though SEM was appropriate for this study, the model did not capture the complex dynamic relationships that exist among the variables. Including the perspectives of multiple stakeholders, such as suppliers, could uncover the challenges faced by organizations to manage cost pressures against sustainability efforts (tensions). Additionally, designers could provide insights into how designers create value for sustainable fashion that consumers find worthy of purchase. Triangulation of consumer data with data from suppliers, retailers, manufacturers, and distributors, and others could reveal important interdependencies within the fashion supply chain that are currently invisible to consumers.

One major limitation of this study is the potential for sampling bias due to the use of intercept surveys conducted in shopping malls. Such locations attract active fashion consumers; yet, these locations tend to attract urban, middle-class consumers who do not necessarily reflect all sustainability-focused consumers. Future studies should actively attempt to balance the sample by conducting online surveys in locations such as social media and sustainability-focused blogs, as well as community-based outreach through local independent designers. A second limitation with this study is the reliance on self-reported survey data. In sustainability-related research, social desirability bias is often a limitation because consumers will provide overly favorable responses due to socially desirable norms. To overcome this limitation, future studies can incorporate procedural and/or statistical remedies such as the social desirability scale (SDS). Further, the study could validate self-reported data with observational data or data collected from purchasing behavior, which can help to validate the reported consumer engagement. A final limitation of the current study is that it did not capture the emotional dimension in measuring multidimensional engagement sustainably. Most studies on engagement lack the measurement of the emotional dimension, which is critical for a holistic perspective of the phenomenon. Future studies need to be conducted to capture the multifaceted nature of consumer engagement with sustainable fashion by incorporating the cognitive, emotional, and behavioral dimensions into the framework.

Future research could investigate other influences on sustainable fashion consumption, including cultural variation in sustainable fashion adoption and the roles of trust, brand loyalty, and social influence. Future studies may also employ a longitudinal design to investigate long-term change in willingness to sustainability, consumer engagement, and brand performance. In order to improve performance analysis from the different stakeholder perspectives, future studies should use multiple data sources, such as from the organizations, suppliers, industry experts, and media. Using more complex SEM models, such as tests of mediated relationships among multiple stakeholders, could provide a better understanding of the relationships between collaborative innovation, consumer engagement, and brand performance in sustainability. Conducting studies in different countries, such as in Western markets like the US, could improve the generalizability of findings.

## 7. Conclusion

The findings also revealed that PCI has a positive and significant effect on PBP, through direct effect, as well as through indirect effect through PBSF and CWASF. PBP is one of the main determinants of consumer adoption of sustainable fashion, and enhancing perceptions of benefits, i.e., PCI is critical to the success of brands in the sustainable fashion industry, and contributes to sustainable growth in the sustainable future. The results have implications for sustainable fashion in emerging markets, such as Thailand, where sustainable fashion is starting to attract the attention of local consumers and retailers. Ways to achieve successful, sustainable fashion in emerging markets need more innovative consumer engagement approaches. Therefore, this study revealed that incorporating PCI efforts with consumer engagement, i.e., CWASF strategies is a winning formula to attain PBP and sustainability innovation. This combines dual strategic benefits for companies and contributes to advancing sustainable innovation for future growth.

## Supporting information

S1 File Item Correlation Matrix. Available at: https://doi.org/10.5281/zenodo.2044163.
(XLSX)

S2 File Appendix. (DOCX)
