# Supplementary material for: Collaborative innovation in sustainable fashion: A consumer-centric perspective on adoption and brand performance
Source: PLoS One. 2026 Jun 11;21(6):e0337902. doi: 10.1371/journal.pone.0337902 (PMC13258014; doi:10.1371/journal.pone.0337902)
Supplement: S2 File — (DOCX) [file pone.0337902.s002.docx]

**Appendix 1**

Instructions: This survey is designed to understand your views and behaviors related to sustainable fashion. Please answer each section carefully. The survey will take approximately 5-10 minutes to complete.

Section 1: Screening Questions

Purpose: To determine eligibility for the study based on experience with sustainable fashion.

1. Are you familiar with sustainable fashion concepts (e.g., eco-friendly materials, ethical labor practices)?

a) Yes

b) No (If no, please skip to the end of the questionnaire. Thank you for your time.)

1. Have you ever purchased or seriously considered purchasing sustainable fashion products (e.g., clothing, accessories)?

a) Yes

b) No (If no, please skip to the end of the questionnaire. Thank you for your time.)

Section 2: Demographics

1. Gender

a) Male

b) Female

c) Prefer not to say

1. Age
   - a) 20-29
   - b) 30-39
   - c) 40-49
   - d) 50 and above
2. Highest Level of Education Completed

a) Below high school

b) High school diploma or equivalent

c) Bachelor's degree

d) Graduate degree

1. Annual Income

a) Less than ฿30,000 (approx. $850)

b) ฿30,000 - ฿59,999 (approx. $850 - $1,700)

c) ฿60,000 - ฿99,999 (approx. $1,700 - $2,800)

d) ฿100,000 and above (approx. $2,800 and above)

1. How would you describe your typical shopping behavior?

a) Primarily online

b) Primarily in-store

c) A mix of both

Section 3: Measurement Constructs

Instructions: Please indicate your level of agreement with each of the following statements on a scale of 1 to 10, where 1 = Strongly Disagree and 10 = Strongly Agree.

1. Perceived Collaborative Innovation (PIC)

*(Based on Wang & Hu, 2020; Swink, 2006)*

- PCI1: Sustainable fashion brands I purchase from frequently collaborate with other companies to innovate.
- PCI2: Sustainable fashion brands I support often use external ideas to improve their products.
- PCI3: Sustainable fashion brands I follow are known for co-creating with their customers.

2. Perceived Benefits of Sustainable Fashion (PBSF)

*(Based on Niinimäki, 2010; Zhang et al., 2024)*

- PBSF1: I believe that purchasing sustainable fashion products reduces environmental impact.
- PBSF2: Buying sustainable fashion supports socially responsible practices.
- PBSF3: Sustainable fashion products offer long-term economic savings.

3. Consumer Willingness to Adopt Sustainable Fashion (CWASF)

*(Based on Ajzen, 1991; Bly et al., 2015;* *Pandey & Yadav, 2023 )*

- CWASF1: I am willing to pay more for sustainable fashion products.
- CWASF2: I prefer to buy sustainable fashion over conventional fashion.
- CWASF3: I actively look for sustainable fashion options when shopping.

4. Perceived Brand Performance (PBP)

*(Based on Liu et al., 2020; Todeschini et al., 2017)*

- PBP1: Sustainable fashion brands that I support have a strong market presence and a good reputation.
- PBP2: I am satisfied with the quality and experience of purchasing from sustainable fashion brands.
- PBP3: I intend to continue purchasing from my preferred sustainable fashion brands.
